# Editorial: Immunity to Human Fungal Pathogens: Mechanisms of Host Recognition, Protection, Pathology, and Fungal Interference

**DOI:** 10.3389/fimmu.2018.02337

**Published:** 2018-10-15

**Authors:** Steven P. Templeton, Amariliz Rivera, Bernard Hube, Ilse D. Jacobsen

**Affiliations:** ^1^Department of Microbiology and Immunology, Indiana University School of Medicine—Terre Haute, Terre Haute, IN, United States; ^2^Center for Immunity and Inflammation, New Jersey Medical School, Rutgers—The State University of New Jersey, Newark, NJ, United States; ^3^Department of Pediatrics, New Jersey Medical School, Rutgers—The State University of New Jersey, Newark, NJ, United States; ^4^Department of Microbial Pathogenicity Mechanisms, Leibniz Institute for Natural Product Research and Infection Biology—Hans Knöll Institute, Jena, Germany; ^5^Institute of Microbiology, Friedrich Schiller University, Jena, Germany; ^6^Research Group Microbial Immunology, Leibniz Institute for Natural Product Research and Infection Biology—Hans Knöll Institute, Jena, Germany

**Keywords:** human fungal pathogens, fungal infection, antifungal immunity, fungal pathology, immune recognition

Fungi are eukaryotic heterotrophs present in virtually every environment, with potentially more than 5 million individual species (1). Despite this impressive biodiversity, only about 300 species are capable of causing disease in humans (2). The most successful human pathogens among these share the ability to grow at the physiologic temperature of endothermic vertebrates and consequently colonize or infect only susceptible hosts (3). Immune-competent humans are largely resistant to fungal infections that throughout much of human history were considered rare and remained poorly understood. However, since 1980, the prevalence of opportunistic fungal diseases has steadily increased in parallel with increases in individuals with acquired immune deficiencies or those receiving immune suppressive or myeloablative therapies (4–6). Worse yet, current options for antifungal therapies remain limited and the continued emergence of resistant strains threatens to further erode antifungal drug efficacy (7, 8). The pressing need for novel therapies has thus resulted in increasing interest in studies in fungal biology and host-fungal interactions that may identify novel antifungal targets or alternative antifungal therapies.

The constant exposure of humans to both commensal and environmental fungi requires a competent immune system for tolerance, protection, and/or clearance, while limiting collateral damage caused by excessive or detrimental inflammation. Innate responses to fungal pathogens are initiated by fungal component recognition via an array of pathogen-associated molecular pattern recognition receptors (PRRs) (9–11). Recognition of fungal ligands by these receptors initiates a cascade of signaling events that result in activation of inflammatory cytokine and chemokine expression, driving recruitment and activation of innate phagocytic cells such as neutrophils and monocytes to the site of infection. Dendritic cells take up fungal particles in this cytokine-rich microenvironment, integrate these activating signals through PRRs and cytokine/chemokine receptors, migrate to site-draining lymph nodes, and subsequently activate naïve fungal-specific T cells. In this manner, early fungal recognition and inflammation provide critical signals that drive adaptive antifungal immunity. However, significant variation encountered by the host innate immune system, and co-evolutionary adaptation of pathogenic fungi, drive diverse disease outcomes ranging from tolerance, clearance and resolution to dissemination and excessive inflammation.

Increased understanding of these host-fungal interactions and the mechanisms that favor protective immunity over immune pathology could provide targets for novel therapeutic approaches that complement the limited repertoire of existing antifungal drugs. The aim of this research topic is to explore the host and fungal pathways that program innate and adaptive immunity and the immune cells, molecules, and regulatory pathways that comprise protective or detrimental responses to fungal exposure or infection. Researchers from 15 countries in North and South America, Europe, Asia, and Australia contributed 36 review and original research articles that cover a wide range of fungal pathogens, disease models, and effector and regulatory cell and molecular pathways of host immune responses to fungal exposure and infection. Here, we present an outline of the findings, perspectives, and reviews contained in this research topic.

The major class of pattern recognition receptors, the C-type lectin receptors (CLRs), including molecules critical for the initiation of inflammation and the development of adaptive immunity to fungi, are reviewed by Tang et al.. Although the important role of the β-1,3-glucan receptor dectin-1 in antifungal immunity is well appreciated, the α-mannan-binding dectin-2 and dectin-3 also influence responses to fungal exposure and infection and their roles are less clear. In support of these studies, Preite et al. show that dectin-2 and dectin-3 mediate antifungal activity and cytokine secretion of human plasmacytoid dendritic cells (pDCs) in response to the endemic dimorphic fungal pathogen *Paracoccidioides brasiliensis* via a pathway mediated by the CLR-associated signaling molecule Syk. In addition, de Castro et al. show that mouse bone marrow-derived macrophages (BMMs) and DCs (BMDCs) produce IL-1β and IL-18 in response to *Fonsecaea pedrosoi*, the main causative agent of chromoblastomycosis, in a dectin−1, −2, and −3-dependent manner. A role for the NOD-Like Receptor P3 (NLRP3), an intracellular protein complex that controls activation of inflammatory caspases and cytokine production, is shown by Feriotti et al. as important for the development of protective immunity to pulmonary infection with *P. brasiliensis*. β-glucan stimulation of NLRP3 inflammasome-mediated IL-1β secretion in B cells reported by Ali et al. shows innate antifungal cytokine production in an adaptive lymphocytes that were also capable of producing IgM in an NLRP3-dependent manner.

In contrast to the protective role for the NLRP3 inflammasome in invasive infection with the filamentous opportunistic mold *Aspergillus fumigatus* (12), Gresnigt et al. report that NOD1-deficient mice exhibit increased protection from invasive aspergillosis compared to wild-type controls. Another potential fungal PRR is the fibrinogen-binding domain receptor FIBCD1, and Schoeler Jepsen et al. demonstrate suppression of IL-8 secretion, mucin production, and airway inflammatory gene transcription in FIBCD1-transfected human lung epithelial cells in response to *A. fumigatus*. Lung epithelial cells are shown by Hayes et al. to produce IL-8 and MCP-1 in response to the *Alternaria* allergen Alt a 1 in a TLR2/4-dependent manner. Collectively, these studies support fungal pattern recognition by an increasing number of receptor families with diverse roles in the development of protective or detrimental immunity to fungal exposure and infection.

PRR-expressing tissue-resident macrophages are part of the first line of defense against fungal infections (reviewed by Xu and Shinohara). Circulating monocytes are subsequently recruited to sites of infection in response to inflammatory signals produced by tissue-resident macrophages. Tóth et al. report that the exposure of hyphae of the dematiaceous mold *Curvularia lunata* to human THP-1 monocytes resulted in increased inflammatory IL-8 and regulatory/anti-inflammatory IL-10, suggesting a possible mechanism for the ability of this species to cause chronic infections in immune competent individuals. Landgraf et al. report that dihydrolipoyl dehydrogenase secreted by *P. brasiliensis* may act as an exoantigen that enhances macrophage phagocytosis. Mukaremera et al. report that hypha of the yeast *Candida albicans* induced low levels of cytokine secretion from human monocytes, with the highest levels from yeast and intermediate levels from pseudohypha, and cell wall mannan depletion partially reversed these responses. Using fluorescence yeast cell wall-labeling to measure yeast division within macrophages, Dagher et al. show that activation of the tyrosine kinase Syk is critical for macrophage control of phagocytosed *C. glabrata*. Together, these studies provide new insights into the roles for monocytes and macrophages in induction and regulation of antifungal inflammation and fungal clearance.

Inflammatory cytokine production by macrophages leads to localized inflammation with production of chemokines that attract additional innate immune cells. Caffrey-Carr et al. report that antifungal protection and recruitment of neutrophils and eosinophils in response to *A. fumigatus* inhalation is dependent on the lipid inflammatory mediator Leukotriene β4. Clark et al. show that a key mediator of chromatin decondensation, protein deaminase 4, and the complement receptor CR3, are critical for neutrophil extracellular trap formation in response to *A. fumigatus*, while hyphal killing required only CR3. The role of innate lymphoid natural killer (NK) cells in innate antifungal immunity is reviewed by Schmidt et al.. These works further illustrate diverse mechanisms and roles for innate myeloid and lymphoid cells in antifungal immunity.

Dendritic cells (DCs) are multifaceted professional antigen presenting cells that integrate antigen uptake and local inflammatory signals in order to effectively prime adaptive antifungal immune responses upon migration to site-draining lymph nodes (9). The role of type I interferon-producing plasmacytoid DCs in antifungal immunity in the context of chronic HIV infection is reviewed by Maldonado and Fitzgerald-Bocarsly. Xu et al. investigated the role of macrophage receptor with collagenous structure (MARCO) on pDC recruitment and induction of antifungal adaptive immunity to *C. neoformans* infection, and report that expression of MARCO promoted recruitment of lymph node DCs and skewed local and systemic T helper cell responses away from protective Th1 responses and toward non-protective Th2 responses. Wong et al. demonstrate a role for leucine-rich repeat kinase 2 (LRRK2) in translocation of NFAT to the nucleus in DCs in the early stages of the non-canonical autophagic response to *A. fumigatus* conidia, thus connecting LRRK2 with NFAT-mediated IL-2 expression in early antifungal immunity. Hellmann et al. compared immune responses to *A. fumigatus* in human and mouse DCs, macrophages, and neutrophils, and observed that human DCs exhibited more significant increases in maturation markers and phagocytic ability than murine DCs, while murine neutrophils and macrophages displayed more reactive oxygen species production after *A. fumigatus* exposure. Collectively, these works provide support for additional mediators of DC function that shape antifungal adaptive immunity, and suggest distinct roles for these cells in human fungal disease and experimental animal models.

After DCs migrate to draining lymph nodes, they present fungal antigen to naïve T cells, inducing antifungal adaptive immunity. This process is influenced by cytokines in the microenvironment. Formation of the antifungal Th17 subset of T helper cells is promoted by the inflammatory cytokines IL-6 and IL-23, and Tristão et al. report that these cytokines, along with the IL-17 receptor A, were necessary for protective lung granuloma formation in mice infected with *P. brasiliensis*. IL-1α/β also influence adaptive immune responses to fungi, such as the dimorphic *Cryptococcus* spp., as Shourian et al. observed that IL-1 receptor type 1-deficient mice had increased cryptococcal burden, eosinophilia, M2 macrophage polarization, and recruitment of CD4+IL-13+ T cells, with a concomitant reduction in pro-inflammatory Th1 and Th17 cytokines. However, excessive inflammation and Th1 activation in *C. neoformans* infection is also detrimental, and was reported by Fa et al. to be mediated by the cell death regulatory genes FADD (Fas-associated death domain) and RIP3K (receptor interacting protein kinase 3). The results of these studies support roles for Th1 and Th17 responses in antifungal immunity, as well as a requirement for regulation of cell death to limit excessive inflammation.

Cell and molecular regulation of immunity occurs at multiple levels from induction to resolution of immune responses. An enzyme that limits available tryptophan and thus dampens immune cell proliferation and effector function, indoleamine 2,3-dioxygenase (IDO), is produced by mammalian hosts and the fungal pathogen *A. fumigatus*; findings that shed light on this host-pathogen dynamic are reviewed by Choera et al.. De Araújo et al. report that DCs from an infected *P. brasiliensis*-susceptible mouse strain exhibit IDO activity and promote inflammation, while DCs from resistant mice phosphorylate IDO and promote a tolerogenic phenotype. The same group further compared *P. brasiliensis* infection in IDO-deficient and wild-type mice, and observed increased mortality, fungal burden, histopathology, and Th17 cell recruitment and activation in IDO-deficient mice with concomitant decreases in Th1 and Treg cells de Araújo et al.. In addition to the enzymatic activity and signaling by IDO, antifungal immune responses are regulated at the post-transcriptional level by microRNAs (miRNAs); these molecules and their associated pathways are reviewed by Croston et al.. Zimmerman et al. report that patients with autoimmune polyendocrinopathy-candidiasis-ectodermal dystrophy (APECED) exhibit decreased levels of the signaling molecule STAT1, in contrast with patients with STAT1 gain-of-function mutations, despite similar disease phenotypes. These works highlight findings of enzymatic, post-translational, and signal transduction in the regulation of antifungal immunity and fungal disease susceptibility.

Although spore/hyphal recognition by innate cells drives the development of immunity to fungal pathogens, host-fungal pathogen interactions are also considered at the macroscopic level of microbial communities (e.g., biofilms) and host tissues. Kernien et al. review host immune recognition of fungal biofilms and biofilm properties that inhibit host recognition and clearance. Zhang et al. explore how immune interactions with respiratory and gastrointestinal fungal microbiota contribute to chronic airway inflammatory disease. Sparber and LeibundGut-Landmann more specifically discuss host immune responses to skin-colonizing *Malassezia* species, yeasts that are involved in a variety of skin disorders. Finally, Casadevall et al. review the evidence that *C. neoformans* mediates host damage at the molecular, cellular, tissue, and organism levels, in some instances with formation of large fungal masses in brain tissue. These reviews highlight emerging areas of fungal immunology that consider fungal interactions with the host immune system beyond the level of cell and molecular recognition.

Despite significant advances in our understanding of host immunity to fungal exposure and infection, treatment of fungal diseases has not progressed beyond the use of a limited repertoire of antifungal drugs that are rendered increasingly ineffective by emerging fungal resistance. Since appropriate antifungal immunity is critical for protection from fungal disease, complementary therapies that target immune pathways represent areas of considerable interest for fungal immunologists. Elsegeiny et al. review studies of immune pathology in cryptococcal infection and discuss the need for immune targeting therapies that suppress immune-mediated damage without further compromising host protection. Van Dyke et al. demonstrate that combining non-lethal *C. neoformans* challenge with local IFN-γ production elicits an immune response that protects mice from subsequent lethal infection. Tso et al. summarize efforts and obstacles in the development of anti-*Candida* vaccines, and discuss the potential use of trained immunity in the development of strategies against opportunistic fungal infections. Finally, Kumaresan et al. review the development of CD8+ T cell therapy for the control of invasive fungal infections, with a focus on the use of chimeric antigen receptor (CAR) T cells that target β-glucan. These reviews underscore the importance of current and future efforts to enhance immune protection while limiting immune pathology in patients that may not respond to traditional antifungal therapies.

Collectively, the studies described in original research and review articles in this topic provide optimism for the future of antifungal immune therapy. Recent advances by fungal immunologists have greatly increased our understanding of the basic mechanisms of innate immune recognition, inflammation, adaptive immunity, and regulation of antifungal immune responses at molecular, cell, tissue, and organismal levels. We hope these articles will stimulate further research with the ultimate goal of improving outcomes for patients with fungal diseases.

## Author contributions

All authors listed have made a substantial, direct and intellectual contribution to the work, and approved it for publication.

### Conflict of interest statement

The authors declare that the research was conducted in the absence of any commercial or financial relationships that could be construed as a potential conflict of interest.

## References

[1] O'brien HE Parrent JL Jackson JA Moncalvo JM Vilgalys R. Fungal community analysis by large-scale sequencing of environmental samples. Appl Environ Microbiol. (2005) 71:5544–50. 10.1128/AEM.71.9.5544-5550.2005 16151147 PMC1214672

[2] Taylor LH Latham SM Woolhouse ME. Risk factors for human disease emergence. Philos Trans R Soc Lond B Biol Sci. (2001) 356:983–9. 10.1098/rstb.2001.0888 11516376 PMC1088493

[3] Robert VA Casadevall A. Vertebrate endothermy restricts most fungi as potential pathogens. J Infect Dis. (2009) 200:1623–6. 10.1086/644642 19827944

[4] Fridkin SK Jarvis WR. Epidemiology of nosocomial fungal infections. Clin Microbiol Rev. (1996) 9:499–511. 8894349 10.1128/cmr.9.4.499 PMC172907

[5] Richardson M Lass-Florl C. Changing epidemiology of systemic fungal infections. Clin Microbiol Infect. (2008) 14 (Suppl. 4):5–24. 10.1111/j.1469-0691.2008.01978.x 18430126

[6] Lehrnbecher T Frank C Engels K Kriener S Groll AH Schwabe D. Trends in the postmortem epidemiology of invasive fungal infections at a university hospital. J Infect. (2010) 61:259–65. 10.1016/j.jinf.2010.06.018 20624423

[7] Ostrosky-Zeichner L Casadevall A Galgiani JN Odds FC Rex JH. An insight into the antifungal pipeline: selected new molecules and beyond. Nat Rev Drug Discov. (2010) 9:719–27. 10.1038/nrd3074 20725094

[8] Fisher MC Hawkins NJ Sanglard D Gurr SJ. Worldwide emergence of resistance to antifungal drugs challenges human health and food security. Science (2018) 360:739–42. 10.1126/science.aap7999 29773744

[9] Wuthrich M Deepe GSJr Klein B. Adaptive immunity to fungi. Annu Rev Immunol. (2012) 30:115–48. 10.1146/annurev-immunol-020711-074958 22224780 PMC3584681

[10] Plato A Hardison SE Brown GD. Pattern recognition receptors in antifungal immunity. Semin Immunopathol. (2015) 37:97–106. 10.1007/s00281-014-0462-4 25420452 PMC4326652

[11] Stappers MHT Clark AE Aimanianda V Bidula S Reid DM Asamaphan P. Recognition of DHN-melanin by a C-type lectin receptor is required for immunity to Aspergillus. Nature (2018) 555:382–6. 10.1038/nature25974 29489751 PMC5857201

[12] Karki R Man SM Malireddi RKS Gurung P Vogel P Lamkanfi M. Concerted activation of the AIM2 and NLRP3 inflammasomes orchestrates host protection against Aspergillus infection. Cell Host Microbe (2015) 17:357–68. 10.1016/j.chom.2015.01.006 25704009 PMC4359672

